# The last low whispers revisited: a reply to Sulmasy on palliative sedation

**DOI:** 10.1007/s11017-026-09762-5

**Published:** 2026-06-03

**Authors:** Harrison Lee

**Affiliations:** University of Mississippi, Oxford, USA

**Keywords:** Terminal sedation, Palliative sedation, Double effect, Timothy E. Quill, Daniel P. Sulmasy, Value of consciousness

## Abstract

Many ethicists who have opposed euthanasia have nonetheless approved of various practices which have been believed to hasten, or risk hastening, the death of terminally ill and suffering patients. For instance, they have approved of a practice which I shall call ‘end-of-life sedation,’ or ‘EOL sedation.’ This practice involves administering drugs with sedating effects to relieve suffering at the end of life. Some have believed that EOL sedation risks hastening death by suppressing respiration in certain cases. The main difference, according to these opponents of euthanasia, is that whereas euthanasia by definition involves an intention to kill, EOL sedation does not. At most, it is argued, EOL sedation foreseeably but unintentionally hastens, or risks hastening death as a byproduct of relieving the patient’s suffering while they are still alive. As such, it has been argued that EOL sedation is supported by the rule of double effect (RDE). In his paper, “The last low whispers of our dead,” Daniel P. Sulmasy raises objections to a specific form of EOL sedation called ‘palliative sedation,’ focusing especially on two variants of that practice which have been defended by Timothy Quill and colleagues. Palliative sedation involves intentionally sedating a patient as a means of relieving their suffering rather than foreseeably sedating them as a byproduct of targeting specific symptoms. Sulmasy argues that palliative sedation, so defined, is neither supported by RDE nor permissible. Here it is argued that Sulmasy’s criticisms of this practice, and of the specific variants of it which he singles out for special criticism, are unsuccessful.

## Introduction

Many ethicists who have opposed euthanasia have nonetheless approved of various practices which have been believed to hasten, or risk hastening, the death of terminally ill and suffering patients. For instance, they have approved of a cluster of practices that involve administering drugs with sedating effects to relieve suffering at the end of life, typically until death. Some have believed that these practices risk hastening death by suppressing respiration in certain cases. However, there are significant differences between the practices in this cluster. Some aim to reduce consciousness and others to eliminate it entirely, while still others do not aim to reduce consciousness at all and only reduce it as a foreseen byproduct of interventions aimed at targeting specific symptoms. In spite of the significant differences between these procedures, I will use the term ‘end-of-life sedation’ (or ‘EOL sedation’) as an umbrella term to refer to all of them, introducing further terminology later on to distinguish between the variants among them. It is controversial whether EOL sedation ever hastens death when administered responsibly. Nonetheless, many opponents of euthanasia have argued that EOL sedation can be permissible even if it hastens death. The main difference, according to them, is that whereas euthanasia by definition involves an intention to kill, EOL sedation does not. At most, it is argued, EOL sedation foreseeably but unintentionally hastens, or risks hastening death as a byproduct of relieving the patient’s suffering while they are still alive. As such, it has been argued that EOL sedation is supported by the rule of double effect (RDE) [1–4]. RDE (elsewhere called the principle of double effect (PDE) or the doctrine of double effect (DDE)) has been formulated in many ways. However, a common version of it maintains, essentially, that (i) it is impermissible to intentionally cause harm or evil even as a means of achieving a greater good, and that (ii) it can sometimes be permissible to cause harm or evil foreseeably as long as one does not cause it intentionally (cf. [1–8]).^1^

In his paper, “The last low whispers of our dead,” Daniel P. Sulmasy distinguishes between various forms of EOL sedation and argues that while some of them are ethically permissible, others are not. Specifically, he takes aim at a form of the practice which has been called ‘palliative sedation,’ focusing especially on two variants of that practice which have been defended by Timothy Quill and colleagues [1]. The defining feature of palliative sedation as I employ that term here is that it involves intentionally sedating a patient as a means of relieving their suffering rather than foreseeably sedating them as a byproduct of targeting specific symptoms. Sulmasy argues that palliative sedation, so defined, is neither supported by RDE nor permissible [1]. Here it is argued that Sulmasy’s criticisms of this practice, and of the specific variants of it which he singles out for special criticism, are unsuccessful.

## Background

Euthanasia has been defined as the practice whereby a physician intentionally kills a patient in order to relieve their suffering [9, 10]. By contrast, the form of EOL sedation that Sulmasy associates with classical applications of RDE involves administering sedatives to patients with the intention of targeting the neurological bases of suffering. Although these sedatives are administered in high enough doses, or in the right circumstances, to pose a high risk of causing the patient to lose consciousness or die, neither of these consequences is intended ([1], pp. 244–7). I will call this practice ‘classical RDE sedation.’ Sulmasy contends that it is frequently morally justified.

Timothy Quill and colleagues describe and defend two further types of EOL sedation, and both of these are forms of palliative sedation. The first of these involves administering titrated sedatives to intentionally diminish a patient’s consciousness just enough to make their suffering tolerable. According to Quill et al., the physician’s intention is not to cause the patient to completely lose consciousness. Nonetheless, they foresee that they might inadvertently cause this effect ([11], p. 421). Sulmasy calls this practice ‘parsimonious direct sedation’ ([1], pp. 248–50). The second type of palliative sedation that Quill et al. defend involves rapidly increasing sedation over the course of minutes or hours with the intention of causing the patient to lose consciousness until death ([11], p. 421–2). Sulmasy calls this practice ‘sedation to unconsciousness and death,’ or ‘STUD’ ([1], pp. 250–2). Quill et al. grant that both parsimonious direct sedation and STUD risk hastening death by suppressing respiration. But they argue that they are distinct from euthanasia since they do not involve an intention to kill. Thus, they argue that they can be defended on the same grounds as classical RDE sedation ([11], p. 421).

Sulmasy argues that neither parsimonious direct sedation nor STUD is in fact supported by RDE, and that both practices are, in fact, impermissible ([1], pp. 248–50, pp. 250–2). His argument is that consciousness is a basic human good. It is what makes humans capable of the cognitive activities that distinguish them from other animals ([1], pp. 237–9). Sulmasy even goes so far as to say that a patient with diminished consciousness is “less human” for that fact ([1], p. 250). And he identifies various ways in which retaining the capacity for consciousness can be especially beneficial for dying patients. Here he is:

The end of life, as Ira Byock has emphasized, presents positive opportunities for growth [12]. Dying patients have significant spiritual/existential needs—concerns regarding questions of meaning, of value, and of relationship—that are most fully explored through conscious expression [13]. It is only through consciousness that patients can truly retain control over their situations, a possibility that many dying persons report as an important personal value [14]. It is through consciousness that they can participate in medical decision making. Importantly, the dying can also serve as teachers to those who care for them and survive them [15]. Such teaching is best accomplished if they are conscious and have the ability to communicate. ([1], p. 239)

Sulmasy’s point, however, is “emphatically not” that consciousness is a good that must always be preserved at all costs ([1], p. 239]). Rather, he clarifies, it is that the loss of consciousness, whether partial or total, must always be considered a cost. As a result, Sulmasy argues, it is almost always wrong to intentionally diminish someone’s consciousness until death. At least, it is not permitted by RDE, which only permits intentionally causing neutral or good effects, and never permits intentionally causing bad effects even for good ends ([1], p. 239, pp. 249–50). The only exception that Sulmasy admits to the prohibition is when the patient’s physical symptoms become so severe that they “effectively colonize [his] consciousness” ([1], p. 258), precluding any “real possibility” for him to enjoy any of the goods that it typically makes possible ([1], p. 253). By contrast, Sulmasy contends that classical RDE sedation receives genuine support from RDE since it does not involve intentionally causing the patient either to lose consciousness or to die. In this practice, he argues, both of these consequences are occasionally foreseen, but they are never intended. As he puts it,

“The last low whispers of our dead” ought not to be intentionally suppressed, although clinicians must accept that these whispers will one day cease for all patients and should be willing to accept their suppression as an unintended side effect of helping patients in other important ways. ([1], p. 239).

To be sure, Sulmasy acknowledges that humans need to go through periods of diminished consciousness, most obviously in the context of sleep. But he argues that usually, sleep is restorative. While it diminishes one’s abilities to achieve cognitive goals in the short-term, it promotes those abilities overall ([1], p. 238). He also acknowledges that it is permissible to administer general anesthesia in the context of surgeries. But he maintains that the purpose of anesthesia in these contexts is also restorative, since it is to facilitate restorative ends like the removal of an inflamed appendix. By contrast, Sulmasy argues that no restorative end is achieved by diminishing a patient’s consciousness until death. By definition, it does not facilitate a more complete recovery of conscious abilities before death ([1], p. 257).

As I noted in the introduction of this paper, RDE has been formulated in many ways. Some formulations simply maintain that it is worse to cause harm or evil intentionally than it is to cause it foreseeably, leaving open the possibility that it could be permissible to cause harm or evil intentionally if a threshold is triggered, that is, if enough is at stake (see [16–19]). Other formulations are absolutist, maintaining that it can *never* be permissible to intentionally cause harm or evil in pursuit of the greater good [6, 20].^2^ Some “threshold” formulations [18] of RDE and many absolutist formulations [6, 20, 21] build upon these minimal foundations by adding lists of varying lengths of further necessary or jointly sufficient conditions for permissible action or for a particular kind of moral justification. Sulmasy maintains that for an action to be justified by RDE, the following 9 conditions must be met:

1. All reasonable and less risky alternatives have been exhausted.
2. There is one action with at least two foreseeable effects.
3. The act itself is good (or at least neutral).
4. One effect is bad while the other is good.
5. The good effect is the discrete event towards which one is aiming (i.e., one’s intention-in-acting or the end of the act), not one’s further intention (i.e., the end of the agent).
6. These good and bad effects are not mediated by intervening agents, but flow immediately from the act.
7. One foresees the bad effect but intends only the good effect.
8. The bad effect is not the means by which the good effect is accomplished.
9. The act is proportionate in these two senses:
10. The means employed are proportionate to the end (means-end proportionality).
11. The potential benefits are proportionate to the potential harms (end-end proportionality). ([1], p. 243)

Sulmasy maintains that to satisfy condition 2, the merely foreseen or risked effect must be of a different type than the intended effect. Thus, he argues that parsimonious direct sedation does not fulfill this condition, since the intended effect is some degree of unconsciousness and the risked effect is a greater degree of the same effect ([1], pp. 248–9). However, Sulmasy does not explain why the intended effect of an action would need to be of a different type than its risked bad effect for the action to be justified by RDE. A lesser and a greater degree of unconsciousness are distinct effects even if they are the same in type, and an agent could intend to cause the former without intending to cause the latter. Moreover, agents can be harmed by greater degrees of an effect where they would not be harmed by lesser degrees of it. Nor is it standard for formulations of RDE to stipulate that the foreseen and intended effects must be different in type.

Based on my reconstruction of Sulmasy’s argument in this section, however, his main objection to parsimonious direct sedation and STUD seems to be that they violate condition 7. Both aim at reducing the patient’s consciousness without any restorative end in view. However, Sulmasy argues, so reducing a patient’s consciousness harms them. For consciousness is what makes distinctly human, meaningful behavior possible. Thus, Sulmasy argues, when unconsciousness does not restore future consciousness, it is a bad effect, and so cannot be permissibly intended consistently with condition 7. Thus, while there are many formulations of RDE, Sulmasy’s chief objection is that it violates a constraint which all or most formulations share, which is RDE’s core constraint against intentionally causing harm or evil.

Initially, it might appear as though Sulmasy has contradicted himself by arguing against intentionally diminishing consciousness before death while advocating for sedative administration with the intention of relieving suffering during this time frame. Do not the sedatives relieve suffering precisely by diminishing consciousness? And if so, then is not the intention in administering them to diminish consciousness in order to relieve suffering? Sulmasy replies that the purpose of sedatives is not to diminish consciousness but rather to diminish the excitability of neurons when they are hyperexcitable due to disease ([1], p. 259). Diminishing the excitability of these neurons relieves suffering on its own, and not by diminishing consciousness. While it is often impossible to diminish their excitability enough to make a patient’s condition tolerable without causing an “extremely high probability” ([1], p. 247) of diminishing consciousness, diminishing the former is nonetheless a distinct goal from diminishing the latter. As such, one can intend to diminish the former without intending to diminish the latter.

## Argument

### The principle of overall benefit

Sulmasy persuasively argues that losing consciousness at the end of life often harms patients in important ways. It prevents them from being able to engage in various behaviors that can be especially meaningful at the end of life, and Sulmasy insightfully identifies many of these ([1], p. 239). However, losing an infected limb also often prevents patients from being able to engage in meaningful behaviors. And it is implausible to suppose that it is impermissible to amputate a limb to prevent an infection from spreading and killing a patient on the grounds that it would also prevent him from engaging in meaningful behaviors. One plausible explanation of why it is permissible to amputate an infected limb is that, even though it harms the patient in important ways, it redounds to his overall benefit. If this is the correct explanation, then, what Sulmasy needs to show is not just that losing consciousness until death harms patients with refractory suffering in important ways. Rather, he needs to show that it always harms them more than it benefits them, or that it harms them *on balance* or *overall*, apart from the rare cases where their consciousness has been colonized. For if it is permissible to intentionally remove a patient’s limb when doing so benefits him overall, in spite of the fact that it also harms him in important ways by preventing him from being able to engage in meaningful behaviors, it should also be permissible to intentionally suppress a patient’s conscious functions until death when doing so benefits him overall.

To be sure, Sulmasy’s explanation of why it is permissible to amputate infected limbs differs from the one I have provided. His explanation comes from what he calls the ‘canon of holism.’ This principle maintains that it is permissible to “alienate parts or functions of a human being…if necessary to preserve higher functions or the survival of the whole person” ([1], p. 241). The differences between this function-based explanation and the benefit-based one I have provided might be important to the present subject matter. One might think that diminishing or eliminating a patient’s consciousness can benefit him overall by relieving otherwise-refractory suffering while denying that it preserves his higher functions. However, it is difficult to see what might be wrong with diminishing a patient’s consciousness if doing so would be the most effective means of benefiting him. One might argue that it would be wrong due to what Sulmasy calls the “canon of restoration,” which maintains that “[t]he goal of all therapeutic intervention is to restore the patient, as far as possible, to homeostatic equilibrium” ([1], p. 240]). Sulmasy clarifies that homeostatic equilibrium is “a normal, pain-free state” ([1], p. 240); 22–23). However, in the cases of palliative sedation that are primarily relevant to the present discussion, these two components of homeostatic equilibrium—i.e., normal functioning and freedom from pain—cannot both be maximized, since normal conscious functioning facilitates pain. The canon of restoration does not explain why, in these cases, normal conscious functioning must be prioritized at the expense of freedom from pain rather than the other way around. Furthermore, even if homeostatic equilibrium consisted solely of normal functioning, maintaining that it is a canon of therapy that it should always aim to promote such equilibrium would simply push the question a step back. The question would be why this should be considered a canon of therapy if one grants that patients are occasionally benefited by losing normal conscious functioning rather than having it enhanced. Of course, one might deny that patients are ever benefited by losing normal conscious functioning, but in the remainder of the paper I will provide various reasons to believe that they are occasionally benefited by this. For now, the point is simply that amputations are very plausibly justified, when they are justified, not *just* by the fact that they preserve the patient’s higher functions or survival, but also by the fact that they benefit the patient by doing so. What is of fundamental importance is benefiting patients, and although preserving their higher functions might typically be the necessary means of benefiting them, it is at least logically possible that in some cases it might not be. Indeed, suppressing a patient’s conscious functions might even benefit him psychologically if it prevents him from having intensely negative experiences. For the absence of any experience at all might be intrinsically better for or less detrimental to one’s psychological well-being than the presence of such experiences.^3^ These reflections support the following principle, which I shall call the principle of overall benefit.

**The principle of overall benefit:** It is *prima facie* permissible to intentionally alienate parts or functions of a patient when alienating them is the least harmful means of benefiting the patient overall.

One might object that my argument so far has been objectionably consequentialist. Non-consequentialism, it would be observed, prohibits intentionally causing harm or harmful bodily impact even where it is the only available means of promoting the greater good. However, this objection would seem to target the canon of holism almost as squarely as it targets the principle of overall benefit. The canon of holism, like the principle of overall benefit, also permits intentionally removing limbs in amputations, which is a serious *pro tanto* harm. If these harms could not be justified by their role in promoting a patient’s greater good, they could not be justified by their role in preserving his biological functioning either. Furthermore, while non-consequentialists refuse some means of facilitating the greater good, these are typically means that either violate an agent’s autonomy or benefit some agents at the expense of others. The principle of overall benefit, however, condones neither sort of action. Rather, it condones intentionally causing *pro tanto* harm (like the removal of a limb) that benefits one and the same individual overall, implicitly when the patient consents and not otherwise.

It is worth emphasizing that RDE in particular need not be construed as prohibiting such *pro tanto* harms. Arguably, in its most plausible formulations, it prohibits or constrains against intentionally harming individuals (i) in order to more greatly benefit others in aggregate or (ii) for no countervailing reason at all. For example, this second condition would imply that a surgeon may not perform a surgery in order to cause the patient to suffer pain in the recovery process, since she would not thereby comparably benefit *anyone*, but she may nonetheless perform the surgery while foreseeing that she will cause the patient to suffer such pain. So interpreted, RDE does not constrain against harming specific individuals in local ways to benefit the same individuals in a global sense. Thus, the principle of overall benefit is not offered as an alternative to RDE but rather as an additional principle which constrains its reasonable application. Indeed, I do not offer any objections to RDE in this paper, but only to Sulmasy’s use of it to argue against palliative sedation.^4^ Furthermore, the purpose of this subsection is not to raise substantive objections to Sulmasy’s argument, but rather to defend a principle—namely, the principle of overall benefit—that motivates some of the objections I raise in subsequent subsections.

A further objection to the principle of overall benefit would maintain that it implies that it could be permissible to lie to patients when it was beneficial to them. For example, it could be argued that it implies that a physician may falsely tell a Jehovah’s Witness that a surgical debridement would not require an intraoperative blood transfusion to secure her consent, thus benefiting her overall. However, the principle of overall benefit only implies that interventions are *prima facie* permissible when they benefit the patient overall. An exception to this permission could reasonably be made for medical lying which, in cases like the one just described, violates patient autonomy. Thus, the principle of overall benefit does not imply that medical lying is permissible. Since I will only be discussing cases where patients or surrogates request palliative sedation, this exception will not impact my argument.^5^

I have not attempted to provide an exhaustive defense of the principle of overall benefit. Rather, I have argued that it yields a plausible way of understanding why medically indicated amputations are permissible, and I have responded to a few likely objections. However, even if one rejects the principle of overall benefit, I believe that it is sufficiently plausible that it should be a matter of interest what follows from it.

### The harmfulness of unconsciousness and the permissibility of classical RDE sedation: A delicate balance

To reiterate, if the principle of overall benefit is true, then Sulmasy needs to show not just that losing consciousness until death harms patients with refractory suffering in important ways, but rather that it always harms them *overall*, except where their consciousness has been colonized. I do not believe that he has shown this. He maintains that permanent unconsciousness (here conceived as unconsciousness which lasts until death) does not enhance future consciousness and so is not restorative ([1], p. 257). But whether or not permanent unconsciousness is restorative in this sense, it can powerfully relieve suffering. And relief from suffering is profoundly beneficial, as Sulmasy acknowledges [pp. 235–7]. Is there any reason to doubt that these benefits can outweigh the harms caused by permanent unconsciousness? One might defend an affirmative answer to this question by appealing to Sulmasy’s claim that someone who lacks consciousness cannot enjoy *any* distinctly human goods [pp. 237–9]. By contrast, someone who has lost an arm due to an amputation can still engage in many meaningful distinctly human behaviors. But if one is just concerned with whether losing consciousness until death can benefit patients overall, what matters primarily is the magnitude of the harms and benefits that it confers, not their type. Whether the benefits are distinctly human appears to be beside the point. If they are substantial enough in a given case, it is unclear why they could not outweigh the harms that result from losing consciousness until death. Indeed, Antony Takla and colleagues have argued that the value of consciousness for an individual depends on its contents, and that the contents of consciousness can be on balance negative for many terminally ill patients at the end of life [24]. Furthermore, patients in deep sedation might retain some distinctly human consciousness [25]. The remaining consciousness that they have might be more peaceful than it would have been otherwise precisely because it has been diminished and so is less burdened by suffering. If so, then diminishing consciousness might benefit patients in distinctly human ways after all. For example, a deeply sedated patient might retain a dim awareness of comforting voices or touch while no longer experiencing the severe suffering that previously burdened their consciousness.

Furthermore, if losing degrees of consciousness until death was always harmful overall (except in cases of “colonized” consciousness), as I have argued Sulmasy’s argument requires, some apparently permissible uses of classical RDE sedation would be threatened. Regardless of whether one diminishes consciousness intentionally or merely foreseeably by some course of treatment, the treatment cannot be justified if it causes more harm than good ([26], pp. 38–9, [27]). However, as we have seen, administering sedatives in classical RDE sedation is believed to be permissible even when doing so poses an “extremely high” risk of diminishing or eliminating the patient’s consciousness ([1], p. 247).

Here, my argument mirrors one that Frances Kamm has used to defend physician-assisted suicide (PAS). Kamm notes that advocates of RDE objections to this practice allow that it can be permissible to administer morphine to relieve suffering in high enough doses that it will foreseeably kill the patient if one’s intention is to relieve suffering and not to hasten death. She argues, however, that this can only be permissible if relief from suffering can be a greater good than continued life. For, she argues, it would be impermissible to relieve a patient’s suffering at the foreseen expense of their continued existence if they were overall harmed by this even if the harm was merely foreseen and not intended. And she argues that since it is permissible or even obligatory for physicians to intentionally cause patients to suffer lesser evils to advance the patients’ medically relevant good, it is permissible or even obligatory for physicians to participate in PAS [27]. In this last step, Kamm is effectively invoking the principle of overall benefit or a strengthened version of it that would make it not just permissible but obligatory for physicians to cause patients to suffer lesser evils to advance their greater good. My argument has been that, for similar reasons, classical RDE sedation is unlikely to be frequently permissible unless relief from suffering can be a greater good than continued consciousness. Thus, I argue that it can be permissible to intentionally cause patients to lose consciousness until death where it would sufficiently reduce their suffering due to the principle of overall benefit.^6^

Of course, one could maintain that it is only permissible to administer sedatives under these circumstances because it is reasonable to expect that, in addition to causing the patient to fall unconscious, administering them will also diminish the excitability of the patient’s hyperexcitable neurons, as Sulmasy observes [1], p. 259]. One could argue that, by diminishing the excitability of neurons in such states, sedatives relieve some amount of additional suffering that they do not relieve by diminishing the patient’s consciousness. And, one could argue, it is only because they relieve this additional amount of suffering that they benefit the patient overall. Otherwise, it would be argued, they would not diminish the patient’s suffering significantly enough to counterbalance the harmful effects that they cause by diminishing his consciousness. Indeed, one potentially significant difference between PAS and palliative sedation is that whereas death completely eliminates suffering in PAS, diminishing a patient’s consciousness in palliative sedation might not. But this argument is based on relatively precise normative and clinical estimates. Namely, it is based on relatively precise estimates of how much patients are harmed by losing degrees of consciousness relative to how much they are benefited by having their suffering relieved, how much of their suffering sedatives relieve by diminishing their consciousness, and how much of their additional suffering sedatives relieve by diminishing the excitability of their neurons. To warrant belief, these estimates would require a rigorous defense that Sulmasy does not appear to have provided.

One might reply that what makes classical RDE sedation permissible when it will almost certainly cause unconsciousness is that there is a chance, however small, that it will not cause this. This potentially small chance of relieving suffering without causing unconsciousness, it would be argued, is what makes the expected impact of classical RDE sedation beneficial overall. It is, of course, possible to justify risky procedures when they have the potential to confer important benefits that cannot be achieved in less risky ways. But recall, Sulmasy’s argument requires that permanent unconsciousness is so harmful that it can *never* benefit a patient overall (except where his consciousness has been colonized) even if it is the only available means of relieving severe suffering. If permanent unconsciousness is so harmful, then it is surely surprising if an extremely high risk of causing a patient to fall unconscious until death can be counterbalanced by a very small chance of relieving suffering without causing him to fall unconscious. In other words, for these risks to be counterbalanced by the prospect of these benefits, the amount that one is harmed by permanent unconsciousness would need to fall within a relatively narrow range. That amount could neither be too low to support the claim that permanent unconsciousness is *always* on balance harmful nor too high to support the claim that its harmfulness can be overridden by a very small chance of relieving suffering without causing unconsciousness. The contention that the harmfulness of permanent unconsciousness lies within this narrow range strikes me as ad hoc and it would require further argument.

Moreover, even where classical RDE sedation does not very substantially risk causing unconsciousness per se, it is virtually certain, in many cases, to at least diminish consciousness. It seems unlikely that the extraordinarily small chance of relieving suffering without even diminishing consciousness could render the expected impact on the patient overall beneficial if permanently diminished consciousness is so harmful that it can never be overall beneficial no matter how much suffering it relieves. Finally, the suggestion that classical RDE sedation is justified by the (occasionally very small) chance that it will not cause permanent unconsciousness suggests that it is impermissible to administer sedatives when doing so will certainly cause permanent unconsciousness. However, it is counterintuitive to suppose that it would not be permissible to administer sedatives under these conditions even if it was needed to relieve extreme suffering, and even if one’s intention was not to cause unconsciousness but rather to diminish the excitability of the patient’s neurons.

None of this is to deny that there is an important ethical distinction between causing harm intentionally and causing it foreseeably. Again, I do not raise any objections to RDE in this paper, but only to Sulmasy’s use of it to argue against parsimonious direct sedation and STUD. I grant that if permanent unconsciousness was always overall harmful, then it would be wrong to cause it intentionally, and that it might be acceptable to cause it foreseeably. Nor do I argue that unconsciousness is caused intentionally in classical RDE sedation, though this has been argued elsewhere [29, 30]. Rather, my argument has been that on the assumptions needed to motivate Sulmasy’s objections to parsimonious direct sedation and STUD, classical RDE sedation can only be beneficial overall provided that the harmfulness of permanent unconsciousness falls within a narrow range. Its harmfulness must be moderate enough that even an extremely high likelihood of causing it can be outweighed by the expected benefits of classical RDE sedation, but severe enough that it can never be overall beneficial on its own in spite of the fact that it powerfully relieves suffering. To establish that the harmfulness of permanent unconsciousness lies within this range, Sulmasy would need to provide further arguments.

One objection to the arguments of this section would come from the New Natural Law (NNL) tradition. This tradition maintains that human flourishing is composed of various “basic goods” including (but not being limited to) life and health, knowledge, aesthetic experience, and play ([31], p. 77). These goods are basic in the sense that their value does not derive from the role that they play in facilitating further goods. The NNL tradition maintains that these goods are “incommensurable.” This means that none of them is more valuable than any other, nor does any play a more important role in human flourishing than any other. As a result, it is argued that it would be wrong to intentionally sacrifice any of them for the sake of another. Such a sacrifice would betray a false judgment that the good pursued was more valuable than the one sacrificed ([31], pp. 82–6). It could be argued that consciousness is also a basic good, and thus, that it can never be intentionally sacrificed to eliminate suffering.^7^

However, if this objection is meant to target the principle of overall benefit, it falls short. If it was successful, it would show that ‘sacrificing’ consciousness by diminishing it cannot benefit the individual overall, since it is incommensurable with any goods for which it might be sacrificed. But the principle of overall benefit does not claim or imply that diminishing consciousness can benefit the individual overall. Rather, it implies that *if* diminishing consciousness can benefit an individual overall, *then* it is permissible for a medical professional to so intentionally diminish it. One could deny the antecedent of this conditional while affirming the conditional as a whole. More importantly, however, it is implausible that consciousness is incommensurable with other goods in the radical way presupposed by the currently envisaged NNL-based objection to my arguments, because one can be benefited overall by sleeping. If the disvalue of unconsciousness could not be compared to the value of its benefits, it could not benefit one overall. And as Sulmasy acknowledges, it is obviously permissible to intentionally cause unconsciousness during normal sleep and anesthesia for restorative procedures. For this reason, Sulmasy himself would likely deny that consciousness is incommensurable with other basic goods. Additionally, if consciousness was radically incommensurable with other goods, one could not be benefited overall by classical RDE sedation if it caused unconsciousness. For the benefits of this procedure would not outweigh the harm that it caused by diminishing consciousness, since they could not be compared. Furthermore, it has been convincingly argued elsewhere that basic goods are not incommensurable in the radical sense required by the presently envisaged NNL objection [32–34]. Finally, I will offer further reasons to believe that we can be overall benefited by unconsciousness at the end of life in particular in the subsections that follow.

### The value of unconsciousness during surgery

I believe that Sulmasy misunderstands why it is obligatory to intentionally suppress patients’ consciousness during surgical procedures. He contends that the reason why this is permissible is that it facilitates restorative benefits. For example, he suggests, one could not perform an appendectomy without first suppressing the patient’s consciousness, since it would cause excruciating pain ([1], pp. 256–7]). However, this argument seems to equivocate on ‘could.’ While it would be unethical to perform an appendectomy without administering anesthesia if it was available, it would not be impossible. Surgeons could restrain the patient to prevent him from interfering with the procedure. And the reason why it would be unethical for the surgeons to forego administering anesthesia is that it would cause the patient to suffer terribly during the procedure. Thus, the reason why they are obligated to administer anesthesia prior to surgery is precisely that it profoundly benefits the patient by causing him to be unconscious and thus preventing him from suffering excruciating pain.^8^ It is not that the loss of consciousness will benefit the patient at some point in the future when he regains consciousness, but rather that it benefits him then and there, while he is unconscious, by preventing him from suffering excruciating pain.

Sulmasy could reply by arguing that the real reason why unconsciousness benefits the patient is not that it prevents him from suffering during the surgery, but rather that it prevents him from experiencing psychological trauma in the future. However, the reason why one would intuitively be more likely to experience trauma after undergoing surgery without anesthesia is precisely that it would be so harmful to him at the time that it was happening. So, while it is plausibly true that administering anesthesia provides restorative benefits to the patient when he regains consciousness in the future by preventing trauma, it only does so by benefiting him during the surgery while he is unconscious.

To see that anesthesia benefits patients during surgeries, and not just afterward when they regain consciousness, consider the case of a patient who dies during surgery. Of course, such a patient is benefited by being unconscious during the procedure even though it does not lead to restorative benefits for him in the future. In retrospect, his loved ones would not regret the fact that he was unconscious during the surgery on the grounds that it prevented him from exercising his cognitive faculties in meaningful ways at the end of his life. If the surgery was what killed him, his loved ones would of course wish that he had not undergone it, but they would not wish that he had not received anesthesia during surgery if he had to undergo it.

These arguments show that unconsciousness can benefit patients not only by restoring their conscious functioning in the future. In surgery, it also benefits them while they are unconscious by preventing suffering. Thus, *pace* Sulmasy, it cannot be objected that unconsciousness harms patients in parsimonious direct sedation and STUD merely because it does not restore their conscious functioning in the future. Instead, it might benefit them while they remain unconscious by preventing suffering.

### Letting dying patients rest

I argued above that it is not sufficient for Sulmasy to maintain that the permanent absence of consciousness always harms patients. Rather, I argued, he must argue that it always harms patients *on balance* except in the ‘extremely rare’ cases where the patient’s consciousness would otherwise be colonized by the symptoms of disease. But I believe that this position leads to implausible conclusions. To see why, suppose that a patient, Mara, is imminently dying and unconscious, but that her doctors have the technical means to awaken her. Suppose that they are certain that if they do not awaken her, she will remain unconscious until death. And suppose that if they were to awaken her at any time before death, the result would be that she would experience unbearable suffering. Additionally, suppose that Mara’s consciousness would not be completely colonized by the symptoms of disease if she were awakened. She would still be capable of using her consciousness in valuable ways (e.g., by courageously facing her suffering). And finally, suppose that Mara had not communicated in advance whether she would want to be awakened in such a scenario. I submit that if Mara was harmed on balance by being unconscious in this scenario, her doctors would be obligated to awaken her. It would be their duty as healers to do so. But clearly, it is not. In fact, it seems that it would be morally wrong for them to awaken her. And the best explanation of why it would be wrong is that Sulmasy is mistaken. Mara would be all-things-considered severely harmed by regaining consciousness. She is much better off unconscious than she would be conscious. Even if it might be valuable for her to courageously face her suffering, one can easily imagine that the value of her doing so would be outstripped by the harm of overwhelming suffering.

One way to resist this argument would be to maintain that Mara would likely not want to be awakened. Thus, the reason that it would be wrong to awaken her is not that it would harm her but rather that it would violate her presumed autonomous wishes to the contrary. But why would it be reasonable to presume that she would not want to be awakened? The most plausible answer seems to be that it would harm her. Thus, this autonomy-based explanation seems to presuppose the harm-based one that I have proposed.

Another response would maintain that the reason that Mara’s doctors are not obligated to awaken her derives from the alleged distinction between doing and allowing harm. This distinction is codified by the doctrine of doing and allowing (DDA), which maintains that it is worse to cause harm than it is to allow it [36]. This doctrine is used to explain why, for example, it does not seem to be murderous to fail to make life saving donations to persons in need from one’s surplus. One allows, but does not actively cause, the would-be recipients of resources to die [36]. Similarly, it would be argued, while it might be impermissible to harm patients by causing them to fall unconscious, it might be permissible to allow patients to be harmed by allowing them to remain unconscious. But while the DDA might explain why it is permissible to keep surplus resources instead of donating them (assuming it is permissible) there are serious difficulties for extending this reasoning to cases like Mara’s. As I have mentioned, physicians have special duties to heal their patients and prevent them from being harmed. It is because they have such duties that they could not plausibly justify failing to provide routine treatments to cure serious illnesses by pointing out that they only allowed, and did not cause, their patients to be harmed. To be sure, these duties can be overridden by refusals of treatment. For example, Jehovah’s Witnesses have the right to refuse life-saving blood transfusions based on their belief that it is impure to consume blood. But I have stipulated that Mara has not communicated a wish not to be awakened. So, this exception does not apply to her situation. Furthermore, even if the DDA could be successfully invoked to argue that Mara’s doctors are not obligated to awaken her, it could not explain why they are seemingly not even permitted to awaken her. Surely, if Mara was harmed by being unconscious, her doctors would at least be permitted to awaken her. But even this weaker result seems implausible.^9^

I do not argue that if Mara’s doctors refrained from awakening her, then they would necessarily intend to cause or allow her to remain unconscious until death. They could instead merely foresee that she would remain unconscious until death if they refrained from intervening. Nor do I claim that Sulmasy himself would argue that Mara’s doctors would be obligated to awaken her. Since he emphatically denies that consciousness is to be pursued at all costs at the end of life, I doubt that he would argue this. Rather, my argument is as follows: (i) there cannot be an unconditional prohibition against intentionally suppressing consciousness permanently (apart from cases of ‘colonized consciousness’) unless permanent unconsciousness is always harmful overall; (ii) Mara’s case shows that permanent unconsciousness is not always harmful overall, since otherwise her doctors would seemingly be obligated to awaken her; therefore, (iii) there is no unconditional prohibition against intentionally suppressing consciousness permanently, *pace* Sulmasy.

## Conclusion

I have argued that Sulmasy’s objections to parsimonious direct sedation and STUD are unsuccessful. I argued that those objections would succeed only if losing consciousness until death always harmed patients overall. However, if losing consciousness until death *always* harmed patients overall, then standard instances of classical RDE sedation would likely be impermissible. Furthermore, I argued that Sulmasy’s explanation of the value of unconsciousness during surgery is mistaken, and that the correct explanation instead supports parsimonious direct sedation and STUD. Finally, I argued that if losing consciousness until death always harmed patients overall, as Sulmasy’s argument appears to require, then doctors would be obligated, implausibly, to restore consciousness in terminally ill patients with refractory suffering once they had already lost it ‘naturally.’

However, while I have argued that losing consciousness until death does not always harm patients overall, I have not defended the absurd conclusion that it *never* harms them overall. And Sulmasy has explained in rich detail many of the ways in which it can do so. In cases where losing consciousness would harm patients overall, it remains an interesting question under which conditions, if any, RDE or neighboring principles [37] might be useful in distinguishing the permissibility of causing permanent unconsciousness intentionally from causing it merely foreseeably. None of my arguments suggest that they would not be useful under such conditions.

I will conclude with some remarks about how the arguments of this paper relate to the adjacent debate surrounding the ethics of euthanasia. As I have noted, opponents of euthanasia frequently argue that it violates RDE’s constraint against intentionally causing harm or evil. One of Sulmasy’s important claims that I have not explored here is conditional in form. It is that *if* the RDE argument against euthanasia succeeds, then a similar RDE argument against parsimonious direct sedation and STUD succeeds as well. His reasoning in defense of this conditional claim is, as far as I can tell, not explicit. On my interpretation, however, he suggests that the most plausible reason to believe that there is a prohibition against intending to kill patients to relieve their suffering is that killing them permanently deprives them of consciousness and all of the goods that it affords. But, Sulmasy observes, diminishing and eliminating consciousness until death does this as well ([1], p. 260). Still, one might object that there is a large difference between causing someone to lose moderate degrees of consciousness in parsimonious direct sedation and causing them to lose it completely in STUD or euthanasia. But it at least seems plausible to me—though certainly debatable ([24], pp. 289–90)—that if the RDE argument against euthanasia succeeds, this must be because it is never permissible to intentionally cause patients to permanently lose consciousness completely. But one philosopher’s *modus ponens* is another’s *modus tollens*, and I have argued that the consequent of this latter conditional claim is false. In future research, it would be interesting to explore this possible defense of euthanasia further.

## Footnotes

1 Some language in this paragraph is adapted from Lee 2026.

2 Here, there is ample room for disagreement about what counts as an ‘evil’ for the purposes of RDE analysis. There is also room for disagreement about whether harms that redound to the same individual’s overall benefit count as harms for the purposes of such analysis.

3 Furthermore, in the remainder of the paper, I will argue that it is occasionally possible to benefit someone by suppressing, rather than preserving, their higher cognitive functions. If these arguments succeed, then benefiting someone is not logically equivalent to preserving their higher cognitive functions.

4 I am grateful to an anonymous referee for pressing me to clarify the logical relationship between RDE and the principle of overall benefit.

5 Another objection might allege that the principle of overall benefit implausibly implies that it is acceptable to intentionally humiliate patients in order to benefit them. For example, suppose that you are Jones’s physician, and you are confident that the only way to cause him to stop smoking is to humiliate him, and that he will not stop smoking otherwise, and that if he does not stop smoking, he will likely develop cancer. Then, it might seem as though the principle of overall benefit permits you to humiliate him, since it would be the least harmful means of benefiting him overall. However, it seems to me that in the vast majority of cases, humiliating a patient would carry unacceptable risks. Rather than causing the patient to stop smoking, you might discourage them from seeking further medical treatment. Additionally, it would be psychologically unhealthy for patients to feel humiliated where they are not sufficiently morally responsible for their smoking habit to warrant such an emotional response. If you may not humiliate Jones in the case envisaged above, perhaps this is because humiliating him would have a suboptimal impact on his expected overall well-being after all. Suppose instead that Jones is a member of a radically conservative religious community whose members oppose smoking so strongly that they ostracize anyone who smokes. Thus far, he has managed to keep his smoking habit a secret. Suppose that you, as Jones’s physician, share footage of Jones smoking with his religious community on social media so that they will ostracize him until he quits. One might think that sharing the footage would be wrong even if you believed it would maximize Jones’s expected well-being by forcing him to quit smoking, since it would involve intentionally damaging his reputation. It could be argued that intentionally damaging someone’s reputation violates their rights or dignity even if it benefits them. However, it seems to me that damaging Jones’s reputation would plausibly infringe his autonomy, since he would very likely not consent to having it damaged. If, on the other hand, we suppose that he specifically requested that you inform his community of his smoking habit so that he would have incentive to overcome it, the intuition that it would be wrong for you to do so diminishes significantly if it does not altogether disappear. However, as I have argued previously, the principle of overall benefit does not condone violating a patient’s autonomy to maximize their well-being. One might insist that you should not share the footage of him smoking even if he consents because doing so would harm his reputation so seriously that it would not benefit him overall. But then one would not have a counterexample to the principle of overall benefit, which only implies that you should share the footage if doing so would be sufficiently likely to benefit him overall.

6 My argument also parallels Judith Jarvis Thomson’s rebuttal to the RDE argument against PAS at a basic level. Thomson argues that (i) RDE most plausibly constrains against causing harm, and that (ii) death is often not a harm where PAS is considered [28]. I concur with premise (i) but add that the type of harm at play is overall harm as opposed to “local” harm. And I maintain that unconsciousness is at least occasionally not an overall harm where sedation is considered at the end of life.

7 Note that Sulmasy himself does not claim that consciousness is incommensurable with other basic goods, so I consider this objection merely for completeness. I am grateful to an anonymous referee for pressing me to consider this objection.

8 I note that it is a defining feature of general anesthesia that it renders the patient unconscious [35, p. 199]. It is ostensibly used precisely for this purpose. Furthermore, Sulmasy grants that it is permissible to use general anesthesia to suppress consciousness in surgery [1, p. 257].

9 Perhaps a defender of Sulmasy could argue that Mara’s doctors should indeed restore her consciousness if they could treat her suffering with non-sedating analgesics. To account for this reply, we can stipulate either that such analgesics are contraindicated for some reason or that they are not available. With these stipulations in place, the decision before the doctors is starkly between allowing Mara to remain unconscious until death without suffering or restoring her consciousness at some point before death and causing her to suffer.
